# Prediction of Composite Clinical Outcomes for Childhood Neuroblastoma Using Multi-Omics Data and Machine Learning

**DOI:** 10.3390/ijms26010136

**Published:** 2024-12-27

**Authors:** Panru Wang, Junying Zhang

**Affiliations:** School of Computer Science and Technology, Xidian University, Xi’an 710126, China; swxxclykz@163.com

**Keywords:** childhood neuroblastoma, multi-omics data, two-step feature selection method, composite clinical outcomes, machine learning

## Abstract

Neuroblastoma is a common malignant tumor in childhood that seriously endangers the health and lives of children, making it essential to find effective prognostic markers to accurately predict their clinical outcomes. The development of high-throughput technology in the biomedical field has made it possible to obtain multi-omics data, whose integration can compensate for missing or unreliable information in a single data source. In this study, we integrated clinical data and two omics data, i.e., gene expression and DNA methylation data, to study the prognosis of neuroblastoma. Since the features in omics data are redundant, it is crucial to conduct feature selection on them. We proposed a two-step feature selection (TSFS) method to quickly and accurately select the optimal features, where the first step aims at selecting candidate features and the second step is to remove redundant features among them using our proposed maximal association coefficient (MAC). Our goal is to predict composite clinical outcomes for neuroblastoma patients, i.e., their survival time and vital status at the last follow-up, which was validated to be two inter-correlated tasks. We conducted a series of experiments and evaluated the experimental results using accuracy and AUC (area under the ROC curve) evaluation metrics, which indicated that by the combination of the integration of the three types of data, our proposed TSFS method and a multi-task learning method can synergistically improve the reliability and accuracy of the prediction models.

## 1. Introduction

Neuroblastoma is a malignant pediatric tumor arising from neuroblasts descended from neural crest cells [1,2] that accounts for roughly 10% of all diagnosed pediatric cancers [2] but 15% of all pediatric cancer deaths [3]. The pathogenesis of neuroblastoma is not yet clear, and there is extreme heterogeneity both clinically and biologically [4,5,6,7]. Owing to this heterogeneity, its essential to accurately predict the most likely disease outcome for patients when they were diagnosed with neuroblastoma [8,9], which is an important and meaningful task. Many studies at home and abroad have found that many factors are associated with the prognosis of neuroblastoma [10,11,12,13] such as age at diagnosis, stage of disease, amplification of the N-myc proto-oncogene MYCN, chromosome abnormality, genetic mutation, etc. Accurately predicting the clinical outcomes for neuroblastoma patients not only helps patients know about their life expectancy but also helps clinicians make well-founded decision and further develop appropriate treatments [14,15,16,17].

Some researchers have employed a single type of omics data to study the prognosis of neuroblastoma patients. In genome field, Hidalgo et al. [11] proposed a computational model by using gene expression data to reveal the molecular mechanism of high-risk neuroblastoma and reveal the determinant of survival in neuroblastoma patients. Cangelosi et al. [13] found that hypoxia is a prognostic prediction marker for neuroblastoma by analyzing the gene expression profiles. The aberrant patterns of DNA methylation are a common feature of most cancers [18] and are demonstrated at the single-gene and genome-wide levels of neuroblastoma. Moreover, DNA methylation is related to the occurrence [18,19,20,21,22,23] and prognosis [24,25,26,27,28] of neuroblastoma. Yang et al. [19] discovered that the neuroblastoma occurrence can be inhibited by using demethylating agents to reverse epigenetic changes. Furthermore, genome-wide analysis of DNA methylation [24] revealed that the CpG island methylator phenotype is a strong determinant of poor prognosis in neuroblastoma. For neuroblastoma, methylation profiles are associated with the clinical outcomes of patients [29,30,31]. Furthermore, DNA methylation is related to gene expression [32,33,34] which is a complex process.

Integrating multiple types of data can compensate for missing or unreliable information in any single data source, and multiple sources of evidence pointing to the same result are unlikely to lead to false positives [35]. This can further deepen the understanding of the occurrence and development of diseases and improve the accuracy of early diagnosis and prognostic prediction [36,37,38]. To our knowledge, some researchers have studied the prognosis of patients by integrating multi-omics data. Mihaylov et al. [39] proposed a data integration method for when the data are heterogeneous and weakly correlated that adopts two inter-related integrative approaches, horizontal integration and vertical integration, to integrate clinical, gene expression, and copy number variation and cancer progression data to predict patient survival. Klim et al. [40] proposed a prediction model framework for small datasets that integrates gene expression and copy number variation data to predict the overall survival of neuroblastoma patients. Tranchevent et al. [41] integrated clinical, gene expression, and transcriptome data and proposed a network analysis method in which the weights of the edges in the network are the normalized and rescaled Pearson coefficients between patient pairs. The integrated data are the input of the network and the topology information of the network is used to train the classification model for predicting the clinical outcomes (‘Death from disease’, ‘Disease progression’ and ‘High-risk’) of patients.

Patients with neuroblastoma have different clinical outcomes [42,43]. In this study, we mainly focused on composite clinical outcomes, i.e., patient survival times and vital status at the last follow-up. To the best of our knowledge, some papers studied one of these two clinical outcomes. The overall survival time of a patient [13] was defined as the time (in years) from disease diagnosis to patient death or the last follow-up (if this patient is alive). Some researchers [44,45,46] have studied the overall survival of patients when the occurrence of death from disease occurs by partitioning patients into two categories, dead or alive. In addition, some researchers [47,48], have divided patients into short- and long-term survivors using a 5-year cutoff and studied the overall survival of patients. In conclusion, these papers studied the 5-year survival times or vital statuses of patients. The survival time of a patient is not equivalent to his/her vital status, and there are four different combinations. A patient’s survival time may be less than 5 years and his/her vital status is still alive, indicating that he/she may survive a longer time in the future. Similarity, a patient’s survival time can more than 5 years but he/she has died, indicating that he/she has an actual and known survival time. In both cases, the survival time and vital status of a patient are in the two categories respectively. Additionally, there are two other cases: a patient’s survival time is less than 5 years and he/she has died or a patient’s survival time is more than 5 years and he/she is still alive. In these two cases, both clinical outcomes are in the same category.

The event we are interested in is a composite clinical outcome of 5-year survival time and vital status (i.e., death or not) for neuroblastoma patients, which constitute two binary classification tasks. We employed a Pearson coefficient between the labels of these two tasks as a quantitative indicator of task correlation [49,50]. The Pearson coefficient between the labels of the survival time and vital status is 0.85, which indicates that the two tasks are inter-correlated. Consequently, we employed a multi-task learning method to solve these two tasks simultaneously. Multi-task learning is a machine learning method based on shared representation that puts multiple related tasks together for learning where different tasks share all or part of the model parameters, which alleviates the demand for data volume to a certain extent. Multi-task learning is a widely used learning paradigm [51,52,53] that is considered as an algorithm-level integration strategy [54]. Multi-task learning has been applied in the field of cancer prognostic study. Shao et al. [55] proposed a multi-task learning framework to study the joint diagnosis and prognosis of cancers for identifying their related features. Maggio et al. [45] proposed a deep learning framework (Concatenated Diagnostic-Relapse Prognostic, CDRP) based on multi-task learning, which obtained more accurate risk stratification to choose appropriate treatment strategies to improve prognosis.

**Figure 1:**
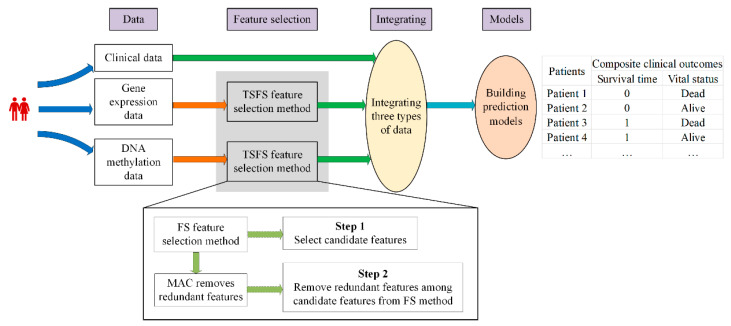
The overall workflow of our study.

The reliability and performance of predicting clinical outcomes can be improved. Although there have been many papers studying clinical outcomes, they aimed at a single clinical outcome, whereas we studied composite clinical outcomes. Because of the high redundancy of omics data and the two inter-correlated tasks of the survival time and vital status, the combination of feature selection and multi-task learning methods seems to be a good solution to solve the problems of prognostic prediction in neuroblastoma. The overall workflow of this study is shown in Figure 1. It is divided into four steps: data, feature selection, integrating, and models. The major contributions of this study are summarized as follows: (1) we built a framework that predicts a composite clinical outcome of 5-year survival time and vital status for neuroblastoma patients by integrating three types of data, i.e., clinical, gene expression, and DNA methylation data, which improves the reliability and accuracy of the prognostic prediction models; (2) we proposed a two-step feature selection (TSFS) method can quickly and accurately select the available features for developing prediction models; and (3) we identified some reliable markers and employed them to build the survival time and vital status prediction models.

## 2. Results and Discussion

### 2.1. Evaluation Metrics

Previously, we have indicated that we studied two binary classification problems. We employed accuracy and AUC (area under the ROC curve) evaluation metrics to evaluate the model performance. It is necessary to understand the confusion matrix before introducing evaluation metrics. For a binary classification problem, the confusion matrix is shown in Table 1.

The confusion matrix summarizes the results of classification prediction according to the real category and the predicted category. As shown in Table 1, *TP* is true positive, which denotes the number of those samples that are predicted to be positive in positive samples; *FN* is false negative, which denotes the number of those samples are predicted to be negative in positive samples; *TN* is true negative, which denotes the number of those samples that are predicted to be negative in negative samples; and *FP* is false positive, which denotes the number of those samples that are predicted to be positive in negative samples. The commonly used accuracy (ACC) evaluation metric is shown in Equation (1).

(1) $$ACC=\frac{TP+TN}{TP+FN+FP+TN}$$

Additionally, the AUC is also a widely used evaluation metric to evaluate the performance of the classification model. The value of the AUC is from 0 to 1. The larger the AUC value, the better the performance of the classification model.

### 2.2. Single-Task Learning Method for Building Prediction Models

In this section, we aim to prove the effectiveness of the TSFS and the reliability brought by the integration of three types of data and the features related to survival time and vital status. Firstly, we employed the single-step feature selection method (FS, described in Section 3.2) and the two-step feature selection method (TSFS, described in Section 3.4) to conduct feature selection. Meanwhile, we integrated three types of data to build the survival time and vital status prediction models by using SVM classifier that is very friendly to small samples. The SVM classifier [56,57,58] has been applied in the field of multi-omics data.

For the clinical data, we referred to previous research [44] and directly employed the three clinical features age at diagnosis in days, INSS stage, and MYCN status to build the survival time prediction model. We employed one clinical feature, age at diagnosis in days, to build the vital status prediction model. Additionally, we conducted min–max standardization to age in the clinical data and conducted one-hot code to INSS stage and MYCN status. For the gene expression and DNA methylation data, we employed the FS and TSFS methods, respectively, to select the optimal features for building the survival time and vital status prediction models. We directly used the selected gene expression and DNA methylation data to build these two prediction models. For the two prognostic prediction models, the hyperparameters *α* (described in Section 3.3, α belongs to [0, 1]) and *Rth* (described in Section 3.4, *Rth* belongs to [0, 1]) used in the TSFS method are empirically set as shown in Table 2. The two hyperparameters are determined according to the prediction results that were obtained by using 5-fold cross-validation.

**Figure 2:**
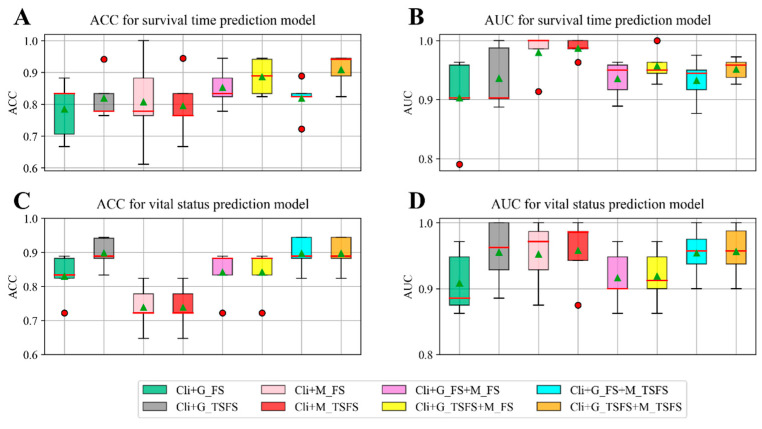
Comparing the classifier performance of each prediction model under eight different types of data. The red circles represent outliers. The green triangles represent the mean of the performance. The red lines represent the median of the performance. (**A**) ACC for the survival time prediction model; (**B**) AUC for the survival time prediction model; (**C**) ACC for the vital status prediction model; (**D**) AUC for the vital status prediction model. Each panel shows the performance for one of the eight types of data; the box plots show the performance distribution over five test sets.

**Figure 3:**
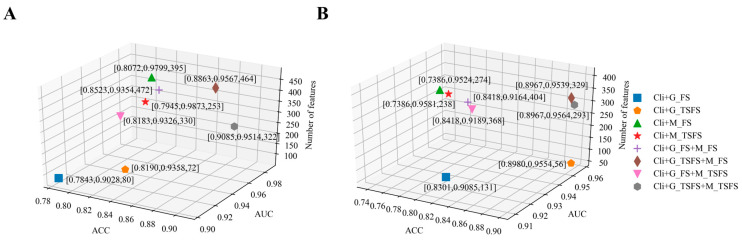
The experimental results of the two prediction models built by the SVM classifier. (**A**) The experimental results of the eight survival time prediction models built by the SVM classifier. (**B**) The experimental results of the eight vital status prediction models built by the SVM classifier. The legend on the right of figure shows the eight different data types for building models.

To demonstrate the validity of the TSFS method and the reliability of the integration of three types of data, we conducted eight different experiments to build the two prediction models. We selected a collection of potential prognosis divers with varying functions and roles in the prognosis of neuroblastoma. We divided the data into five train/test splits, as well as training and testing the model on each split. The classifier performance on each test split is shown in the form of box plots in Figure 2 in order to observe the performance distribution. The average of the results obtained from five test splits is adopted to show the model performance, as shown in Figure 3. In the legends of Figure 2 and Figure 3, ‘Cli’ represents the selected clinical data, ‘G_FS’ represents the selected gene expression data by the FS method, ‘G_TSFS’ represents the selected gene expression data by the TSFS method, ‘M_FS’ represents the selected DNA methylation data by the FS method, and ‘M_TSFS’ represents the selected DNA methylation data by the TSFS method. In addition, the symbol ‘+’ represents the early integration method, i.e., the omics data is integrated by the method of concatenation.

It can be seen from Figure 2 that the classifier performances of the ACC and AUC on ‘Cli+G_TSFS’ are better than those of ‘Cli+G_FS’ for these two prognostic prediction models. Although the classifier performances of the ACC and AUC on ‘Cli+M_TSFS’ are slightly better than those of ‘Cli+M_FS’, the features for building prediction models are reduced, indicating that we should remove the redundant features. These results verify the validity of the TSFS method in building prediction models. Additionally, the ACC of ‘Cli+G_TSFS’ is better than that of ‘Cli+M_TSFS’ but the AUC of ‘Cli+M_TSFS’ is better than ‘Cli+G_TSFS’ for both the survival time and vital status prediction models, indicating that the DNA methylation data provided better model reliability but poorer model performance than the gene expression data. Previously, DNA methylation data have been proven to be useful for the prognosis of neuroblastoma. Moreover, the classifier performances of the ACC and AUC on ‘Cli+G_TSFS+M_TSFS’ are slightly better than those of ‘Cli+G_TSFS’; we argue that simple multi-omics integration (concatenation-based data integration method) may not be able to effectively mine hidden or complementary information between different omics data.

The model performance, i.e., the ACC and AUC, as well as the number of features used to build survival time and vital status prediction models, are shown in Figure 3. In Figure 3A,B, these eight computational models are presented by using eight different markers. There is a list of numbers next to each marker that sequentially represent ACC, AUC, and the number of features of this specific model. For example, the list [0.7843, 0.9028, 80] in Figure 3A shows that 0.7843 is the ACC of this model, 0.9028 is the AUC of this model, and 80 is number of features for building this model. In Figure 3A,B, the model near to the bottom right is better than the other models, i.e., the model has larger ACC and AUC values and fewer features compared with other models.

It should be noted in Figure 3A that, compared with the survival time prediction model built by ‘Cli+G_TSFS+M_FS’, although the AUC of the model built by ‘Cli+G_TSFS+M_TSFS’ is slightly lower at about 0.5%, the ACC is higher, at about 2%, and the number of features is greatly reduced. After comprehensive consideration, we employed ‘Cli+G_TSFS+M_TSFS’ to build the survival time prediction model. It should be noted in Figure 3B that, when building the vital status prediction models by ‘Cli+G_TSFS+M_TSFS’ and ‘Cli+G_TSFS’, the differences in the ACC and AUC between these two models were 0.1%. When the differences in both the ACC and AUC between two models are small, we prefer the model with a larger AUC. Consequently, we employed ‘Cli+G_TSFS+M_TSFS’ to build the vital status prediction model. Finally, for the survival time prediction model, we integrated 3 clinical, 69 gene, and 250 methylation features to build this model. For the vital status prediction model, we integrated 1 clinical, 55 gene, and 237 methylation features to build this model.

We show the experimental results of the survival time and vital status prediction models built by integrating the clinical data and the multi-omics data selected according to the FS and TSFS methods in Figure 3A,B. From Figure 3A,B, we can obtain the following results: (1) the features selected by the TSFS method are better than FS method and (2) the integration of multiple types of data can provide reliable information compared with a single data source, which is of great significance in studying cancer prognosis.

### 2.3. Multi-Task Learning Method for Building Prediction Models

**Figure 4:**
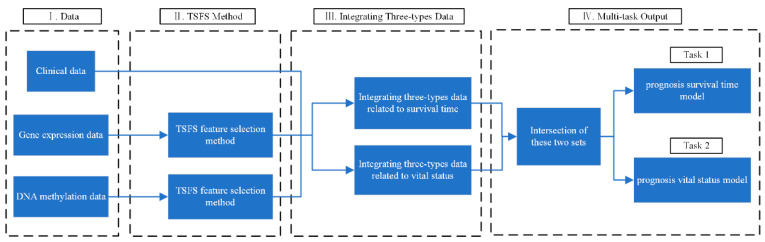
The research framework for the prognostic prediction in neuroblastoma is divided into 4 steps. I. Data. Clinical data and two types of omics data, gene expression and DNA methylation data, are used to predict composite clinical outcomes for neuroblastoma patients. II. TSFS method. The first step aims to select candidate features. The second step aims to remove the redundant features among them. III. Integrating three data types. The three selected types of data related to survival time and vital status are integrated by using the concatenation method. IV. Multi-task output. The intersection of two sets obtained in step III is used as the input of the multi-task learning method. Then, the results of two tasks are outputted simultaneously.

Since the two tasks of predicting survival time and vital status are inter-correlated and the number of samples is small, we employed the multi-task learning method MMoE (Multi-gate Mixture-of-Experts, described in Section 3.5) to solve these two tasks simultaneously. The research framework is shown in Figure 4, and the prediction results are outputted simultaneously during the final modeling.

To select features that are available for both survival time and vital status prediction models, the intersection of the clinical, gene, and methylation features regarding these two models identified in Section 2.2 were used as the input features for multi-task learning. The number of features of single-task learning and that of multi-task learning are shown in Table 3, where ‘SingleOS’ and ‘SingleVS’ represent the survival time and vital status prediction models built by a single-task learning method (SVM classifier) and ‘MultiOSVS’ represents the prediction model built simultaneously by a multi-task learning method (MMoE).

We identified some features that are used in the multi-task learning method, some of which have also been reported in other papers, as shown in Table 4. However, the fact that some biomarkers in this study have not yet been reported does not reflect that they have no effect on prognosis. Various further experiments are needed to verify these unreported biomarkers regarding prognosis.

**Figure 5:**
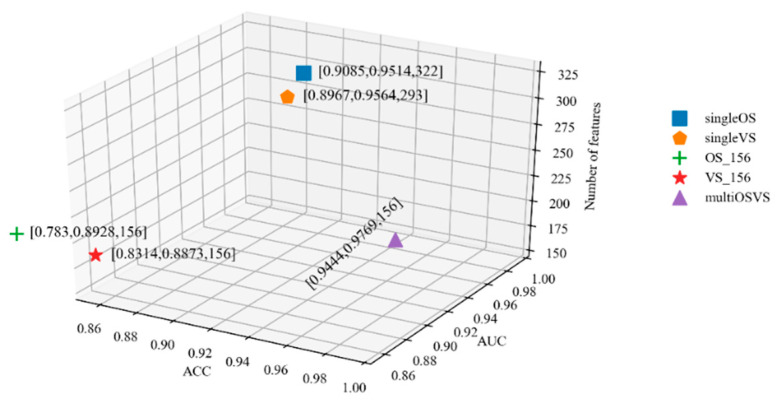
Performance comparison of the prediction models built by single-task and multi-task learning methods.

We employed the MMoE multi-task learning method to build the survival time and vital status prediction models simultaneously and jointly evaluated these two models by the absolute accuracy and AUC. The so-called absolute accuracy refers to the ratio of the number of patients whose predicted survival time and vital status labels are completely consistent with their true labels compared to the total number of patients. We randomly split the dataset into 80% for the training dataset and 20% for the testing dataset. We used five-fold cross validation in training data to obtain the optimal parameters of the MMoE model and tested this specific model in testing data. The experimental results of the survival time and vital status prediction models built by using single-task and multi-task learning methods are shown in Figure 5. As shown in the legend on the right of Figure 5, the five different models are presented by different markers. The meanings represented by ‘singleOS’, ‘singleVS’, and ‘multiOSVS’ are consistent with those in Table 3. In addition, ‘OS_156’ and ‘VS_156’ represent the survival time and vital status prediction models built by the features related to both the survival time and vital status, which are 1 clinical, 4 gene, and 141 DNA methylation features. There is a list of numbers next to each marker that sequentially represent the ACC, AUC, and the number of features of this specific model. For example, the list [0.9444, 0.9769, 156] shows that 0.9444 is the ACC of this model, 0.9769 is the AUC of this model, and 156 is number of features for building this model. In Figure 5, the closer the model is to the bottom right, the better the performance of the model. This signifies that the model has larger ACC and AUC values and fewer features compared with other models.

The ACC and AUC of ‘singleOS’ are better than those of ‘OS_156’. Similarly, the ACC and AUC of ‘singleVS’ are better than those of ‘VS_156’. The comparison results show that the features previously described in Section 2.2 are effective for predicting the survival time or vital status of a patient. Compared with single-task learning, the eliminated gene features in multi-task learning are shown to have an impact on survival time or vital status.

It has been proven that multi-task learning can improve the performance of each task. With the MMoE multi-task learning method, we used features related to both the survival time and vital status to build the prognostic prediction model. As shown in Figure 5, we know that the ACC and AUC of the survival time and vital status prediction models simultaneously built by using multi-task learning method are better than the two models built by using the single-task learning method. Compared with the ‘SingleOS’ and ‘SingleVS’ models, not only are the number of features that are used to build the ‘MultiOSVS’ model reduced but also the performance of this model is improved. This shows that multi-task learning can achieve higher prediction accuracy by using fewer features than single-task learning.

## 3. Materials and Methods

### 3.1. Dataset

**Figure 6:**
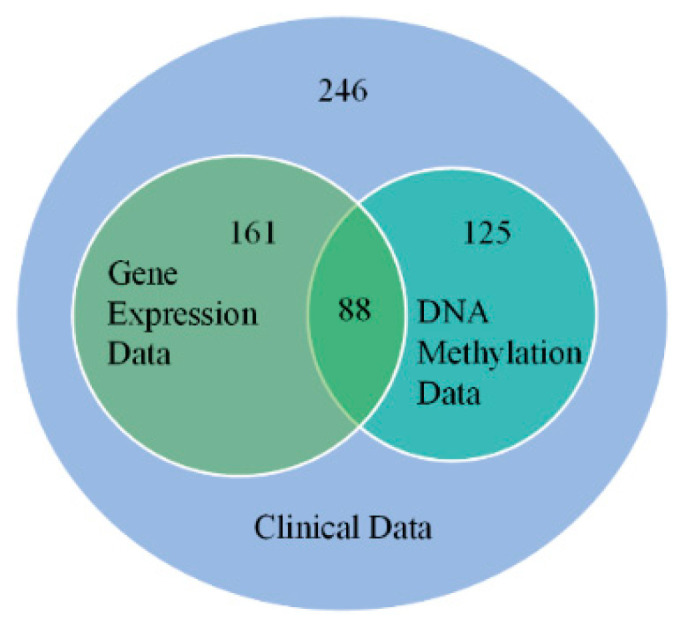
The overlap between the three types of data shown by a Venn diagram.

The neuroblastoma dataset contains clinical and various omics data from the TARGET database (Therapeutically Applicable Research To Generate Effective Treatments, see https://ocg.cancer.gov/programs/target (accessed on 9 November 2020)). In this study, we focused on integrating the clinical data and two types of omics data, i.e., the gene expression and DNA methylation data, to study the prognosis of neuroblastoma patients. We first downloaded these three types of data, as well as their ground-truth survival time and vital status contained in the clinical dataset. We used a Venn diagram to reveal the overlap between the three types of data, as shown in Figure 6. We only employed sample sets with clinical, gene expression, and DNA methylation data, leaving us with a total of 88 samples.

The features of the clinical dataset contain: age at diagnosis in days, INSS stage, MYCN status, and so on. We listed the demographic information of the patients from the neuroblastoma dataset in Table 5. We can know some information: (1) the average age of patients is 3 years old; (2) the number of male patients is 1.38 times higher than that of female patients; (3) 78.41% of patients are White; (4) the INSS stage of 85.23% patients is stage 4; (5) 86.36% of patients are high risk; (6) the number of dead patients is 1.38 times higher than that of alive patients at the last follow-up; and (7) the average survival time is 5.02 years and the median survival time is 4.58 years.

There are 22,985 genes in gene expression dataset, in which the missing data were filled by the mean method. For DNA methylation dataset, all empty features and features with a missing data amount greater than 5% were deleted, and finally 372,843 features remained in which the missing data were also filled using the mean method.

In cancer prognostic research [47,71,72], patients are divided into short-term and long-term survivors according to a set survival threshold, where the threshold is usually set to 3 or 5 years. In this study, the threshold was set to 5 years, the neuroblastoma patients were divided into 46 short-term survivors labeled 0 and 42 long-term survivors labeled 1. For patients, there were two types of vital status at the last follow-up, i.e., dead or alive; there were 51 dead samples labeled 0 and 37 alive samples labeled 1.

### 3.2. FS Feature Selection Method

The Fisher score (FS) [73,74] is a high-efficiency filter feature selection method. Its main idea is that the distance between samples is as small as possible in the same categories and as far as possible in different categories for the features with a strong discrimination performance. The score *S_i_* of the *i*-th feature obtained by the FS method is shown in Equation (2)

(2) $$S_{i}=\frac{\sum_{j=1}^{k}n_{j}{\left(\mu_{ij}-\mu_{i}\right)}^{2}}{\sum_{j=1}^{k}n_{j}{\left(\rho_{ij}\right)}^{2}}$$

where $\mu_{ij}$ and $\rho_{ij}$ are the mean and the variance of the *i*-th feature in the *j*-th class, $n_{j}$ is the number of instances in the *j*-th class, and $\mu_{i}$ is the mean of the *i*-th feature.

The importance of the features is ranked according to the scores of all features. The higher the score, the higher the ranking. We employed the FS method on each piece of omics data and determined the optimal feature number *k* based on the prediction results that were obtained by using 5-fold cross validation. However, the FS feature selection method evaluates the significance of features individually, while ignoring the potential correlation information among them [75]. Consequently, we employed our previously proposed maximal association coefficient (MAC) method [76] to detect the association between any two features among the features selected by the FS method to remove redundant features, which is briefly described in Section 3.3.

### 3.3. Maximal Association Coefficient

**Figure 7:**
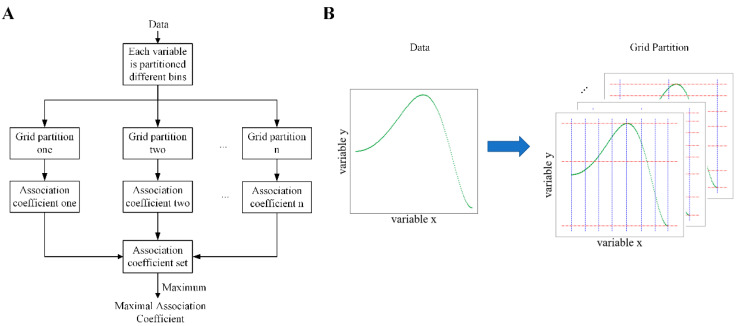
The overview of the proposed maximal association coefficient (MAC). (**A**) The scheme for the MAC. (**B**) The schematic diagram of grid partition. The nonlinear association is composed of some piecewise-linear ones, but no one knows where the breakpoint for connecting two piecewise-linear ones is. Clustering techniques are one of the options to achieve the partition for avoiding the infinite number of partitions caused by a random partition. Afterwards, we employed a simple and commonly used clustering method, K-means, to partition each variable space into different bins. All data can be divided into different grids; the schematic diagram is shown in (**B**). And then, the Pearson coefficient was used to detect the linear association of the data in each grid. The weighted sum obtained by directly using the Pearson coefficient of data in each grid cannot reflect the association between two variables since the Pearson coefficient is between [−1, 1], which causes an offset. Consequently, we applied the absolute value of the Pearson coefficient to detect the linear association of data in each grid. In summary, we employed partition and the Pearson coefficient to measure the association between two variables.

In this section, we will briefly introduce the maximal association coefficient (MAC) that was used to measure the association between two features. The association between them may be nonlinear, which can be partitioned into several piecewise-linear associations. This is a method to use a linear correlation coefficient to measure the nonlinear association between them. The piecewise-linear association can be achieved by a partitioning method. The schematic diagram of the MAC is shown in Figure 7A.

It is necessary to set a maximum number of grids (MG) to avoid infinite grids. The maximum number of grids (MG) is $MG=\max\left\{4,n^{\alpha }\right\}$, where n is data size and α is a hyperparameter and α belongs to [0, 1]. For variable *x* and variable *y*, they are divided into *s* and *t* bins, where *t* is equal to MG/s. The data are divided into s×ts*t grids, where *s* belongs to [2, MG/2], so that we can obtain many different forms of grid partition between two variables. Under each grid partition, an association coefficient (AC) between variable *x* and *y* is calculated based on Equation (3).

(3) $$AC{\left(x,y\right)}_{\left(s,t\right)}=\sum_{i}w_{i}\left|p_{i}\right|$$

In Equation (3), *i* is the *i*-th grid containing data, $w_{i}$ is the weight of *i*-th grid, and ${|p}_{i}|$ is the absolute value of the Pearson coefficient of data in *i*-th grid. The $w_{i}$ can be obtained according to Equation (4).

(4) $$w_{i}=\frac{s_{i}}{\sum_{j}s_{j}}$$

In Equation (4), $s_{i}$ is the area of the *i*-th grid and $\sum_{j}s_{j}$ is the sum of the areas of all grids that contain data. Moreover, in Equation (4), the weight $w_{i}$ is the normalization process to the area, so that the sum of the weights is 1, i.e., $\sum_{i}w_{i}$ is 1.

The association coefficient under each grid partition can be obtained by Equation (2), in which the maximum value of these association coefficients is the maximal association coefficient (MAC), as shown in Equation (5).

(5) $$MAC\left(x,y\right)=\underset{s,t}{\max}\left\{AC{\left(x,y\right)}_{\left(x,t\right)}|s\times t=MG\right\}$$

### 3.4. TSFS Method

**Figure 8:**
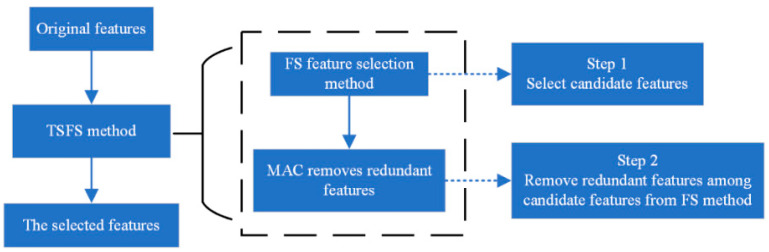
The scheme for the two-step feature selection (TSFS) method. As shown in Figure 8, the TSFS method selects the optimal features in two steps, where the first step is elementary selection and the second step is secondary selection. The combination of the elementary and secondary selections can quickly and accurately conduct feature selection. The novel idea is that the purpose of the elementary selection is to initially screen the candidate features that can be used to build a model; the secondary selection is to refine the features obtained from the elementary selection to obtain the optimal features for modeling. The FS is a high-efficiency feature selection method that can be used as the elementary selection. The MAC can be used to measure the association between two features to mine the potential information between them for revealing the correlation of the two features. Since the MAC can accurately detect an association between two features, it can be used as the secondary selection. In the TSFS method, step 1 is to select the candidate features using the FS feature selection method and step 2 uses the MAC to detect the association among candidate features obtained in the step 1 to achieve the purpose of removing redundant features utilizing the information provided through association strength between them. In this way, the optimal features for downstream classification tasks are selected by the TSFS method.

Considering that it is not only to quickly select candidate features for classifying but also to accurately remove the redundant features between them, we propose a novel two-step feature selection (TSFS) method in this section. The framework of the TSFS method is shown in Figure 8.

**Figure 9:**
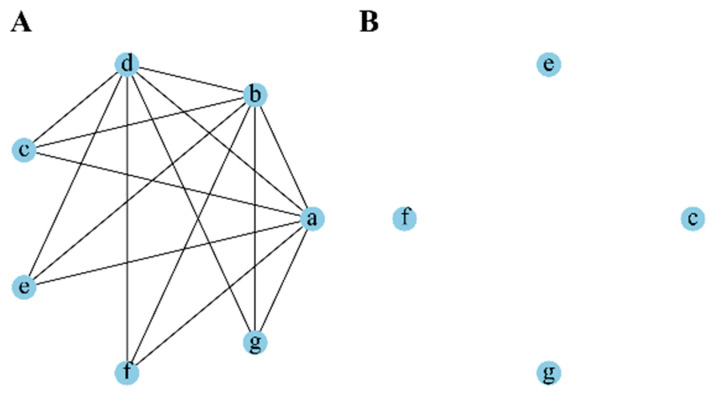
The association between features and redundance threshold (*Rth*). (**A**) The association between features before removing redundant features. (**B**) The remaining features after removing redundant features.

For removing redundant features, we defined a redundance threshold (*Rth*) that is a hyperparameter. If the MAC between two features is equal to or greater than *Rth*, then one of them is a redundant feature. As shown in Figure 9, we revealed the association between features before and after removing redundant features in the form of a schematic diagram. This is an undirected graph, where a node represents a feature, and different letters in the node represent different features. Before removing redundant features, there is an edge between two nodes when the MAC between them is equal to or greater than *Rth*, as shown in Figure 9A. After removing redundant features, the remaining features are shown in Figure 9B.

The adjacency matrix *W* represents the connection relationship between nodes in the graph, where *W_ij_* is the weight of the edge between node *i* and node *j,* as shown in Equation (6)

(6) $$W_{ij}=\left\{\begin{array}{l} 1,\;if\;MAC\left(i,j\right)\geq Rth,\\ 0,\;\;otherwise, \end{array}\;\forall i,j\in R^{n},i\neq j\right.$$

where *i* is the *i*-th feature, *j* is the *j*-th feature, and *MAC*(*i*, *j*) is the maximal association coefficient (MAC) between feature *i* and feature *j* and is equal to or greater than the hyperparameter *Rth*.

The redundant score for each feature can be obtained according to the above adjacency matrix W, as shown in Equation (7), where $W_{ij}$ is obtained according to Equation (5). Afterwards, the purpose of removing redundant features is achieved according to the redundant scores of the features. The obtained redundant scores of all features were sorted in descending order. The higher the feature ranking, the higher the redundancy of the feature. We removed some redundant features based on the prediction results that were obtained by using 5-fold cross validation.

(7) $${Score}_{i}=\sum_{j\neq i}W_{ij}$$

### 3.5. Multi-Task Learning

Multi-task learning [50,77,78,79] is a machine learning method whose goal is to optimize several tasks simultaneously to improve the performance of each task. In this study, we employed a multi-task learning method [50], Multi-gate Mixture-of-Experts (MMoE) proposed by Google, to simultaneously build the survival time and vital status prediction models for neuroblastoma patients. The MMoE considers the commonalities and characteristics of different tasks, thereby reducing the drag between tasks with a weak correlation and improving the sharing between tasks with a strong correlation. The MMoE contains input, gate, shared-bottom expert, top tower, and output layers. The MMoE set up multiple expert networks on the shared-bottom expert layer, each of which is called an expert. Each task corresponds to one gate model. For different tasks, the output of a specific gate represents the probability that different experts are selected. The probability-weighted sum of different experts is obtained and used as the input of a specific tower model to obtain the final output.

## 4. Conclusions

In this study, we proposed a research framework that can quickly and accurately select the optimal features and integrate clinical data and two types of omics data for developing a prediction model with better performance than one with a single data source. Moreover, more and more omics data can be used to test and improve the prediction model due to advances in high-throughput technology. However, there may be a limitation in integrating more omics data since (1) data collection is hard and (2) patients with multiple types of data are few. Nevertheless, researchers can still employ our research framework in their studies no matter how many types of omics data they have.

Accurately predicting the clinical outcomes of neuroblastoma patients can help physicians design personalized treatments. In this study, we aimed to investigate composite clinical outcomes, i.e., survival time and vital status, by integrating clinical data and two types of multi-omics data, i.e., gene expression and DNA methylation data. Owing to the high redundancy of omics data, we proposed a two-step feature selection method (TSFS) to quickly and accurately select the available features for downstream tasks. Ultimately, we identified 1 clinical, 4 gene, and 151 DNA methylation features to build a prediction model. These identified biomarkers may need to be verified by various biological experiments.

As a small sample and inter-correlated multi-task problem, the final prediction model is given via the Multi-gate Mixture-of-Experts (MMoE) multi-task learning method. Consequently, we employed the MMoE method to simultaneously build the survival time and vital status prediction models for patients by integrating three types of data. The clinical outcomes derived from prediction models not only help clinicians to improve the accuracy of predictions but also help doctors pay attention to words when facing patients and their families, which can make them mentally and physically healthy and reduce their mental stress. Moreover, we can know which patients will have an indeterminate survival time according to the predicted vital status, and these patients may survive longer than the predicted results.

## Figures and Tables

**Table 1:** A binary classification confusion matrix. TP: True Positive; FN: False Negative; FP: False Positive; TN: True Negative.

Confusion Matrix		Real	
Predicted		Positive	Negative
	Positive	TP	FP
	Negative	FN	TN

**Table 2:** The hyperparameters of the TSFS method in two prognostic prediction models.

Hyperparameter	Survival Time Prediction Problem		Vital Status Prediction Problem	
	Gene data	methylation data	Gene data	methylation data
*α*	0.7	0.55	0.35	0.6
*Rth*	0.65	0.9	0.2	0.9

**Table 3:** Comparison of the number of features for prediction models built by single-task and multi-task learning methods.

Feature	SingleOS	SingleVS	MultiOSVS
Clinical	3	1	1
Gene	69	55	4
Methylation	250	237	151

**Table 4:** Reports on features related to the prognosis of neuroblastoma.

Name	Category	Description	References
Age	Clinical	Age at diagnosis.	[44]
PLXDC2	Gene expression data	PLXDC2 is the surface receptor of pigment epithelium-derived factor (PEDF); PEDF effects induces cell differentiation and neurite outgrowth.	[59,60]
LRRN2	DNA methylation data	LRRN2 serves as a prognostic marker of NB.	[61]
SFN	DNA methylation data	The methylation of the SFN gene above a defined threshold is a strong and reliable predictor of adverse outcome independently from other prognostic factors.	[62]
FGFR4	DNA methylation data	The FGFR4 is associated with an increased prevalence of neuroblastoma in children.	[63]
MGMT	DNA methylation data	MGMT methylation is a relevant therapeutic target in neuroblastoma.	[64]
IGF2BP3	DNA methylation data	An IGF2BP3 positive coefficient was a risk factor for poor prognosis and the levels of IGF2BP3 and N-myc are positively correlated in NB.	[65,66]
TP73	DNA methylation data	The TP73 gene, also called p73, banded at 1p36.3, is homologue of the TP53 tumor suppressor. TP73 can inhibit cell proliferation and induce apoptosis; a role for TP73 in the development of neuroblastoma could not be completely ruled out. TP73 has been proposed as a candidate tumor suppressor gene involved in neuroblastoma development.	[67,68,69]
NR2E1	DNA methylation data	Elevated expression of NR2E1, also called TLX, in neuroblastoma (NB) correlates with unfavorable prognosis.	[70]
DNA methylation regulates gene expression.			[32,33,34]

**Table 5:** Clinical characteristics of 88 patients with neuroblastoma.

Variables	Categories	Frequency	Percentage (%)
Average age at diagnosis in years: Mean (SD *)	Male	3.01 (2.80)	
	Female	3.03 (1.55)	
	All patients	3.02 (2.36)	
Gender	Male	51	57.95
	Female	37	42.05
Race	White	69	78.41
	Black or African American	9	10.23
	Unknown	7	7.95
	Native Hawaiian or other Pacific Islander	2	2.27
	Asian	1	1.14
INSS stage	Stage 1	12	13.64
	Stage 3	1	1.14
	Stage 4	75	85.23
COG risk group	Low risk	12	13.64
	High risk	76	86.36
Vital status at the last follow-up	Dead	51	57.95
	Alive	37	42.05
Overall survival time of all patients in years: Mean (SD *)	Median survival time	4.58 (3.43)	
	Average survival time	5.02 (3.43)	

* SD: Standard deviation.

## Data Availability

Data is contained within the article or requested from the authors.
